# Psychosocial predictors of delay in receiving mammography results

**DOI:** 10.1192/j.eurpsy.2026.11546

**Published:** 2026-07-31

**Authors:** I. Chaari, A. Samet, W. Abid, Y. Fourti, F. Charfeddine, L. Aribi, N. Messedi, J. Aloulou

**Affiliations:** Psychiatric department “B”, Hedi Chaker University Hospital, Sfax, Tunisia

## Abstract

**Introduction:**

Delays in communicating mammography results may generate anxiety. Psychosocial characteristics may influence how these delays are experienced.

**Objectives:**

To investigate associations between socio-demographics, personality traits, and perceived delay in receiving mammography results.

**Methods:**

We conducted an online survey of the general female population in Tunisia via a sponsored Facebook campaign (December 2023–February 2024). Participants provided the dates of their mammogram and results notification, and the delay in days was subsequently computed. Associations between delay, educational level, socio-economic status, and personality traits (using the Big Five Inventory (BFI-44)), were examined using regression analyses.

**Results:**

A total of 278 women participated. The mean age was 46 years (SD 8.9). Most were married (84.9%) and university educated (78.4%), with two-thirds reporting middle socio-economic status (66.5%). Regarding communication, the median delay was 0 days, with 56% receiving results the same day, 84% within one week, and only 5% after more than one month. The mean delay was 4.8 days (SD 12.3). Lower education was associated with longer perceived delays (β = +2.4 days, p = 0.04). Higher Neuroticism also predicted longer delays (β = +1.8 days, p = 0.03).

**Conclusions:**

In Tunisian women, educational level and neuroticism significantly influenced perceived waiting times. Implementing rapid communication strategies and targeted reassurance may improve the screening experience in vulnerable subgroups.

**Disclosure of Interest:**

None Declared

